# The relationship between cortical glutamate and striatal dopamine in first-episode psychosis: a cross-sectional multimodal PET and magnetic resonance spectroscopy imaging study

**DOI:** 10.1016/S2215-0366(18)30268-2

**Published:** 2018-10

**Authors:** Sameer Jauhar, Robert McCutcheon, Faith Borgan, Mattia Veronese, Matthew Nour, Fiona Pepper, M Rogdaki, James Stone, Alice Egerton, Frederico Turkheimer, Philip McGuire, Oliver D Howes

**Affiliations:** a Department of Psychological Medicine, King's College, London, UK; b Department of Psychosis Studies, King's College, London, UK; c Centre for Neuroimaging Sciences, Institute of Psychiatry, Psychology and Neuroscience, King's College, London, UK; d Early Intervention Psychosis Clinical Academic Group, South London and Maudsley NHS Trust, London, UK; e Psychiatric Imaging Group MRC London Institute of Medical Sciences, Hammersmith Hospital, London, UK; f Institute of Clinical Sciences, Faculty of Medicine, Imperial College, Hammersmith Hospital, London, UK

## Abstract

**Background:**

The pathophysiology of psychosis is incompletely understood. Disruption in cortical glutamatergic signalling causing aberrant striatal dopamine synthesis capacity is a proposed model for psychosis, but has not been tested in vivo. We therefore aimed to test the relationship between cortical glutamate concentrations and striatal dopamine synthesis capacity, and psychotic symptoms.

**Methods:**

In this cross-sectional multimodal imaging study, 28 individuals with first-episode psychosis and 28 healthy controls underwent ^18^F-DOPA PET (measuring striatal dopamine synthesis capacity), and proton magnetic resonance spectroscopy (measuring anterior cingulate cortex glutamate concentrations). Participants were recruited from first-episode psychosis services in London, UK and were required to be in the first episode of a psychotic illness, with no previous illness or treatment episodes. Exclusion criteria for all participants were: history of substantial head trauma, dependence on illicit substances, medical comorbidity (other than minor illnesses), and contraindications to scanning (such as pregnancy). Symptoms were measured using the Positive and Negative Syndrome Scale. The primary endpoint was the relationship between anterior cingulate cortex glutamate concentrations and striatal dopamine synthesis capacity in individuals with their first episode of psychosis as shown by imaging, examined by linear regression. Linear regression was used to examine relationships between measures.

**Findings:**

Glutamate concentrations showed a significant inverse relationship with striatal dopamine synthesis capacity in patients with psychosis (*R*^2^=0·16, p=0·03, β −1·71 × 10^−4^, SE 0·76 × 10^−4^). This relationship remained significant after the addition of age, gender, ethnicity, and medication status to the model (p=0·015). In healthy controls, there was no significant relationship between dopamine and glutamate measures (*R*^2^=0·04, p=0·39). Positive and Negative Syndrome Scale positive psychotic symptoms were positively associated with striatal dopamine synthesis capacity (*R*^2^=0·14, p=0·046, β 2546, SE 1217) and showed an inverse relationship with anterior cingulate glutamate concentrations (*R*^2^=0·16, p=0·03, β −1·71 × 10^−4^, SE 7·63 × 10^−5^). No relationships were seen with negative symptoms (positive symptoms, mean [SD] −18·4 (6·6) negative symptoms, mean [SD] −15·4 [6·1]).

**Interpretation:**

These observations are consistent with the hypothesis that cortical glutamate dysfunction is related to subcortical dopamine synthesis capacity and psychosis. Although the precise mechanistic relationship between cortical glutamate and dopamine in vivo remains unclear, our findings support further studies to test the effect of modulating cortical glutamate in the treatment of psychosis.

**Funding:**

Medical Research Council, Wellcome Trust, Biomedical Research Council, South London and Maudsley NHS Foundation Trust, JMAS Sim Fellowship, Royal College of Physicians (Edinburgh) (SJ).

## Introduction

The dopamine hypothesis of schizophrenia has been one of the most enduring scientific hypotheses within psychiatry. From its origins in the mode of action of antipsychotic drugs, this hypothesis has evolved into a more nuanced model of dopamine dysregulation leading to psychosis.^1^ Theories have incorporated the effect of various genetic and environmental factors on this system, and suggest that glutamatergic dysfunction might underlie striatal dopamine dysregulation at onset of psychosis.^2^ Meta-analytic data from in-vivo molecular imaging (single-photon-emission tomography, single-photon-emission CT, and PET) studies have shown dopamine synthesis capacity is greater in individuals with schizophrenia than in health controls, with an effect size of around 0·7.^3^ Dopamine synthesis capacity has also been found to be directly correlated with symptoms in some studies of people at ultra-high risk^4^ and with established psychosis.^5^

Previous studies using proton magnetic resonance spectroscopy (MRS) to measure brain glutamate concentrations in schizophrenia have produced mixed findings, which could reflect effects of age, illness stage, or antipsychotic medication. Studies reporting glutamate concentrations (as opposed to glutamine, or the sum of glutamine and glutamate, termed Glx) in the frontal cortex are equivocal.^6^ When the relationship between glutamate concentrations and symptom severity has been examined, no consensus has been found, with both positive and negative correlations reported.^7^

**Research in context**

**Evidence before this study**

Animal and drug models of psychosis have suggested a link between cortical glutamate dysfunction and dopamine dysregulation in schizophrenia. We searched PubMed for studies published from database inception until July 3, 2018 that used neuroimaging to investigate both dopamine and glutamate function in psychosis. Search terms were “dopamine”, “glutamate”, and “psychosis”. Previous studies have found a negative correlation between hippocampal glutamate and striatal dopamine measures in individuals at risk of psychosis. In healthy controls, a positive correlation between anterior cingulate cortex glutamate and striatal dopamine measures has been observed. To date, the relationship between cortical glutamate and striatal dopamine has not been directly examined in people with psychotic illness.

**Added value of this study**

Our study of individuals with a diagnosis of first-episode psychosis showed a negative correlation between anterior cingulate cortex glutamate concentration and striatal dopamine synthesis capacity, and that both measures related to PANSS were associated with PANSS positive symptom scores. To the best of our knowledge, this is the first study to demonstrate a relationship between dopaminergic and glutamatergic function and psychotic symptoms in individuals with psychosis.

**Implications of all the available evidence**

The dopaminergic and glutamatergic systems seem to be correlated in patients with first-episode psychosis. The combination of multimodal imaging techniques with longitudinal randomised treatment designs has the potential to advance our understanding in this area.

Glutamatergic projections to the midbrain play a major role in regulating activity of mesostriatal dopamine neurons.^8^ Direct pathways from the prefrontal cortex to the midbrain have an excitatory influence and enhance dopamine release, whereas indirect pathways involving GABAergic interneurons have the opposite effect.^9^ If direct pathways predominate, then lower glutamatergic signalling is expected to lead to lower striatal dopaminergic signalling, whereas if indirect pathways dominate, then lower glutamatergic signalling will disinhibit mesostriatal dopamine neurons, resulting in greater dopaminergic signalling. The possibility that the second of these mechanisms predominates was raised by an in-vivo study in healthy controls, which found an inverse correlation between glutamate in the dorsolateral prefrontal cortex and striatal dopamine synthesis capacity.^10^

It has been proposed that in individuals with schizophrenia, glutamatergic dysfunction in the frontal cortex leads to negative and cognitive symptoms, and, via projections to the midbrain, disinhibits mesostriatal dopamine neurons, resulting in positive psychotic symptoms.^2^ However, to our knowledge, no study has investigated whether striatal dopamine synthesis capacity and cortical glutamate concentrations are correlated in people with psychosis, or if this correlation is related to symptoms.

In this study, we estimated anterior cingulate cortex glutamate concentrations, using proton MRS, and striatal dopamine, using ^18^F-DOPA PET, in individuals presenting with their first episode of psychosis. We hypothesised that anterior cingulate glutamate concentrations would be inversely correlated with striatal dopamine synthesis capacity, and that the severity of psychotic symptoms would correlate directly with dopamine synthesis capacity, and inversely with glutamate concentrations.

## Methods

### Participants

Participants were recruited from first-episode psychosis services in London, UK and were required to be in the first episode of a psychotic illness, with no previous illness or treatment episodes. Participants had to have a diagnosis of a psychotic disorder meeting ICD-10 criteria^11^ and to have experienced psychotic symptoms, defined as a rating of at least moderate severity on one or more of the delusion, hallucination, and persecution items on the Positive and Negative Syndrome Scale (PANSS).^12^ Diagnosis was confirmed by a study psychiatrist (SJ), using a structured instrument (Mini-International Neuropsychiatric Interview).^13^ Inclusion criteria also required people with psychosis to be antipsychotic naive, antipsychotic free for at least 6 weeks, or minimally treated (receiving antipsychotic medication for 2 weeks or less).

Healthy control participants were recruited through local media from the same geographical area as the patients. Inclusion criteria for controls were: no personal history of psychiatric illness (assessed using Mini-International Neuropsychiatric Interview) and no con-current psychotropic medication (through self-report).

Exclusion criteria for all participants were: history of substantial head trauma, dependence on illicit substances, medical comorbidity (other than minor illnesses), and contraindications to scanning (such as pregnancy). Symptoms were measured using PANSS, with raters blinded to imaging results. Age, gender, and ethnicity (white or non-white) were also recorded.

Ethical permission was given by East of England–Cambridge East Ethics Committee, and the Administration of Radioactive Substances Advisory Committee. All participants provided written informed consent to participate.

### PET imaging acquisition and analysis

All participants were asked not to eat or drink (except water), and to refrain from alcohol for 12 h before scan. Imaging data were obtained on a Siemens Biograph 6 HiRez PET scanner (Erlangen, Germany) in 3D mode. 1 h before scan, participants received 400 mg entacapone, a peripheral catechol-*o*-methyl-transferase inhibitor, and 150 mg carbidopa, a peripheral aromatic acid decarboxylase inhibitor, to prevent formation of radiolabelled metabolites that might cross the blood–brain barrier. Participants were positioned in the scanner with the orbitomeatal line parallel to the transaxial plane of the tomograph. Head position was marked and monitored; movement was minimised with a head strap. After acquiring a CT scan for attenuation correction, approximately 150 MBq of ^18^F-DOPA was administered by bolus intravenous injection 30 s after start of PET imaging. PET data were acquired in 32 frames of increasing duration over the 95 min scan (frame intervals: 8 × 15 s, 3 × 60 s, 5 × 120 s, 16 × 300 s). Our primary measure was striatal influx constant (*K*_i_^cer^) for the whole striatum.

Correction for head movement during scan was done by a mutual information algorithm, as described in previous publications.^14^, ^15^ SPM 8^16^ was used to automatically normalise a tracer-specific ^18^F-DOPA template together with the striatal brain atlas as defined by Martinez and colleagues.^17^ *K*_i_^cer^ was calculated using the Patlak–Gjedde graphical approach adapted for a reference tissue input function, used in previous studies by our group.^13^, ^18^

### MRS acquisition

**Figure 1:**
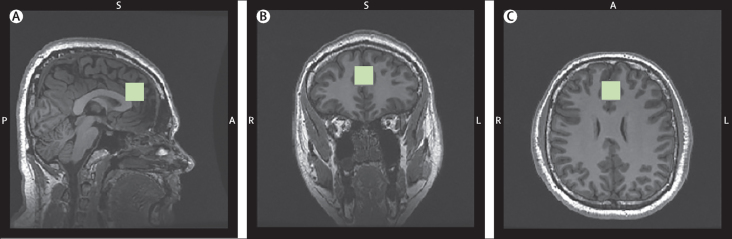
Voxel placement for measurement of anterior cingulate cortex glutamate concentrations

All scans were acquired on a General Electric (Milwaukee, WI, USA) Signa HDxt 3 Tesla MRI scanner. Internal localiser scans were used to establish the anterior commissure–posterior commissure line and interhemispheric angle. Two structural scans were obtained: an axial 2D T2-weighted fast-spin echo scan and an axial fast fluid-attenuated inversion recovery. For the voxel placements, 3D coronal inversion recovery-prepared spoiled-gradient echo scans were acquired, followed by auto prescans for optimisation of water suppression and shimming. Proton MRS spectra were acquired for the anterior cingulate region-of-interest (right–left 20 mm  × anterior–posterior 20 mm  ×  superior–inferior 20 mm). The placement of the anterior cingulate voxel was based on the midline sagittal localiser with the centre of the 20 mm  ×  20 mm  × 20 mm voxel placed 13 mm above the anterior portion of the genu of the corpus callosum, perpendicular to the anterior commissure–posterior commissure line to minimise inclusion of white matter and cerebrospinal fluid (CSF, see figure 1). Finally, proton MRS spectra (Point RESolves Spectroscopy, echo time 30 ms, repetition time 2 s) were obtained through the PROton Brain Examination sequence by General Electric, which includes water suppression.

### MRS analysis

All water-scaled metabolites were analysed with an LC-model 6.3-I0 to estimate concentrations of glutamate.^19^ Spectra were inspected visually and metabolite analyses were restricted to spectra with line width (full-width at half-maximum) ≤0·1 ppm, Cramér–Rao lower bounds for glutamate ≤20%, and signal to noise ratio ≤5. Corrections were applied to account for relative distribution of CSF within the anterior cingulate. In-house scripts were used to identify the relative distribution of white matter, grey matter, and CSF in the 2 cm^2^ voxel prescribed to the anterior cingulate. The following correction was subsequently applied to correct for CSF content within the 2 cm^2^ voxel; where M=raw metabolite value, WM=white matter, and GM=grey matter:

Mcorr=M([WM+GM]×1·22+[CSF×1·55])WM+GM

### Sample size and power calculation

We determined the minimum sample size required to test our primary hypothesis (that there was an inverse relationship between dopamine and glutamate measures). As no previous studies have examined the relationship between dopaminergic and glutamate measures in vivo, in patients with psychosis, we used the one previous study of this relationship in people at risk of psychosis to estimate effect size. This study found a relationship between these measures, with an *R*^2^=0·28 and sample size of 16.^20^ To be conservative, we therefore powered the study to detect an *R*^2^ of at least 0·25. By use of this effect size, the power calculation indicated a sample size of 26 would have more than 80% power to detect a significant relationship between these variables, at p<0·05 (two-tailed).

### Statistical analysis

All statistical analysis was done by use of *R* version 3.3.2 and significance set at p<0·05 (two-tailed). Our primary analysis fitted a linear regression model with striatal dopamine synthesis capacity as the dependent variable, and anterior cingulate cortex glutamate concentrations as predictor. Given the possible effects of age,^21^ sex,^22^, ^23^ and ethnicity^24^ on imaging measures, we did secondary exploratory analyses that adjusted for these demographic factors. Additionally, as antipsychotic treatment could influence imaging measures and symptom severity ratings^25^, ^26^ we added medication status at time of scan (on antipsychotic treatment or drug free) to this model. The same approach was used to investigate whether the relationship between imaging measures and symptom severity measures was influenced by these factors.

### Role of the funding source

The study funders had no role in study design, collection, analysis, or interpretation of data or writing of the manuscript. The corresponding author had full access to all data for the study, and made the decision to submit for publication.

## Results

29 patients completed the study but data from one participant were excluded owing to poor quality proton MRS spectra on visual inspection, leaving 28 patients in the analysis. Demographic details are given in the table; there were no clinically significant differences in age, gender, or ethnicity between patient and control groups. 79% of the patients were men, and the mean age was 25·4 years (table). Mean total PANSS score was 69·2 (SD 19·1), and 25 (89%) of the 28 patients were not taking antipsychotics at the time of scanning (of whom, 12 [48%] had never taken antipsychotics).

**Table:** Demographic details of study participants

		**Patients (n=28)**	**Controls (n=20)**
Sex			
	Male	22 (79%)	13 (65%)
	Female	6 (21%)	7 (35%)
Age, years (mean [SD])		25·4 (3·9)	23·5 (4·1)
Ethnicity			
	White	11	13
	Black	8	2
	Other	9	5
Medication status			
	Antipsychotic naive	12 (43%)	NA
	Minimally treated†	3 (11%)	NA
	Antipsychotic free	13 (46%)	NA
Injected radiolabel activity (MBq)		143·4 (7·5)	153·2 (12·9)
Duration of illness (months)*		23 (28·25)	NA
PANSS			
	Positive	18·4 (6·6)	NA
	Negative	15·4 (6·1)	NA
	General	35·1 (9·0)	NA
	Total	69·2 (19·1)	NA

Data are expressed as n (%) or mean (SD), unless specified. PANSS=Positive and Negative Syndrome Scale.

* Median (IQR). NA=not applicable.

† Receiving antipsychotic medication for 2 weeks or less.

Of the three patients taking antipsychotics at time of scan (minimally treated, for less than 2 weeks), one patient was taking amisulpride 150 mg once daily, one patient was taking amisulpride 200 mg once daily, and one patient was taking risperidone 2 mg once daily. Two patients were taking clonazepam 0·5 mg twice daily, and one patient was taking an antidepressant medication (sertraline 150 mg once daily) at time of scan. When patients were taking treatment, there was no change in medication status between PET and MRS scans.

Median time between PET and MRI scans was 6 days (IQR 2·75–24·25). Some of the PET, but not MRS, data have previously been reported as part of a different analysis.^13^

15 people met ICD-10 criteria for schizophrenia, one for schizophreniform disorder, 11 for bipolar affective disorder, and one for severe depression with psychotic features.

**Figure 2:**
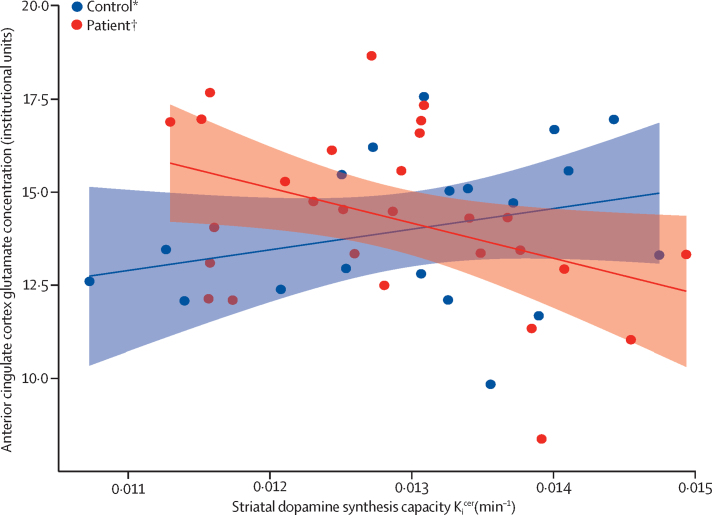
Relationship between anterior cingulate cortex glutamate concentrations and striatal dopamine synthesis capacity *p=0·39. †p=0·03.

We observed an inverse relationship between striatal dopamine synthesis capacity and anterior cingulate glutamate concentrations (β −1·71 × 10^−4^, SE 7·63 × 10^−5^, p=0·03, *R*^2^=0·16, *r*=−0·40) in patients, but not in individuals in the control group (β 1·15 × 10^−4^, SE 1·30 × 10^−4^, p=0·39, *R*^2^=0·04, *r*=0·20; see figure 2). To establish whether the relationship observed in patients could be influenced by confounding variables, we fitted additional regression models, including these variables. In a model including glutamate, age, gender, and ethnicity, glutamate concentrations remained predictive of striatal dopamine synthesis capacity (β −2·03 × 10^−4^, SE 7·70 × 10^−5^, p=0·015). When medication status was included in this model, glutamate concentration remained a significant predictor (β −1·79 × 10^−4^, SE 8·12 × 10^−5^, p=0·039), indicating that antipsychotic medication did not explain the relationship. For the individuals in the control group, including demographic variables did not have any effect on the dopamine–glutamate relationship, which remained non-significant (β 1·38 × 10^−4^, SE 1·45 × 10^−4^, p=0·36).

When Glx, rather than glutamate, was used as a predictor variable in patients, the relationship with dopamine was no longer significant in the initial model (β −9·6 × 10^−5^, SE 6·4 × 10^−5^, p=0·14). The relationship was significant when demographic variables were included (β −1·6 × 10^−4^, SE 6·8 ×  10^−5^, p=0·029), although not when medication status was also added (β −1·4 × 10^−5^, SE 6·9 × 10^−5^, p=0·050). For individuals in the control group, the relationship was not significant for any of the models (p=0·46–0·55). A post-hoc analysis showed that there was a significant difference in the strength of the bivariate dopamine–glutamate correlation between patients and controls (Fisher's r-to-z: z=−2·01, p=0·04).

**Figure 3:**
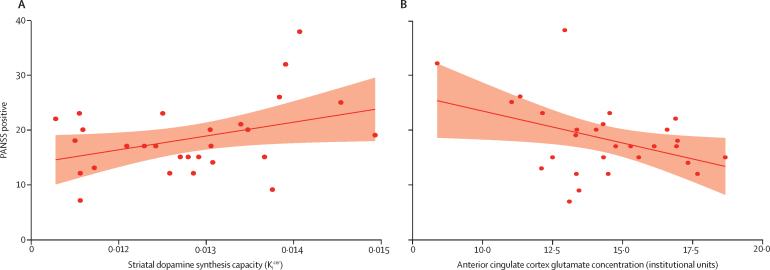
Relationships between positive psychotic symptoms and (A) striatal dopamine synthesis capacity*, and (B) anterior cingulate cortex glutamate concentrations† The red line represents a linear regression where symptom scores are the dependent variable and the imaging measure is the independent variable.*p=0·046. †p=0·03.

We next explored the relationship between dopamine and PANSS-positive psychotic symptoms. The initial model showed a direct relationship between striatal dopamine synthesis capacity and PANSS-positive symptom severity rating (β 2546, SE 1217, p=0·046, *R*^2^=0·14; figure 3). Addition of age, gender, and ethnicity weakened the relationship between dopamine synthesis capacity and positive symptoms (β 2099, SE 1180, p=0·089). Adding medication status slightly strengthened the relationship between dopamine and symptoms, although it remained non-significant (β 2269, SE 1266, p=0·087). We then examined the relationship between cortical glutamate and symptoms. The initial model with glutamate concentrations as the sole predictor of PANSS positive scores showed an inverse relationship (β −1·16, SE 0·51, p=0·03, *R*^2^=0·16). The inclusion of age, gender, and ethnicity strengthened the relationship between glutamate concentrations and positive symptoms (β −1·40, SE 0·44, p=0·0045; figure 3). Adding medication status had no marked effect on the relationship between glutamate and symptoms (β −1·52, SE 0·47, p=0·0037).

There were no significant relationships between negative symptom severity ratings and either glutamate or dopamine measures (glutamate p=0·37, *K*_i_^cer^, p=0·09). A post-hoc analysis showed that there were no statistically significant differences between patients and controls in mean striatal *K*_i_^cer^ (t[46]=−0·37, p=0·71) or anterior cingulate glutamate concentrations (t[46]=0·38, p=0·71).

## Discussion

Our main finding is that an inverse relationship exists between anterior cingulate cortex glutamate concentrations and striatal dopamine synthesis capacity in people with first-episode psychosis. Furthermore, PANSS-positive symptoms were directly related to dopamine synthesis capacity and inversely related to anterior cingulate glutamate concentrations.

Preclinical studies have shown that *N*-methyl-D aspartate receptor blockade, induced using ketamine, increases striatal dopamine concentrations.^27^ Evidence from PET studies in humans also shows that *N*-methyl-D aspartate receptor blockade by ketamine results in dopamine release to a challenge, as measured by the displacement of D_2_ and D_3_ ligands.^28^, ^29^ Furthermore, hippocampal glutamate concentrations have been found to be inversely related to striatal dopamine synthesis capacity in people at risk of psychosis.^20^ Thus, several lines of evidence indicate that alterations in cortical glutamate signalling could lead to disinhibition in striatal dopamine synthesis capacity. Our results extend these previous findings to show, for the first time as far as we are aware, an inverse relationship between cortical glutamate and striatal dopamine in people with psychosis. This correlation indicates that, in individuals with psychosis, those with lower cortical glutamate concentrations show greater striatal dopamine synthesis capacity. It is also possible that the observed relationship is due to striatal dopamine function driving medial prefrontal cortex glutamate function. Longitudinal studies are now required to establish the direction of causality.

Our study extends previous studies by testing the relationship between psychotic symptoms, striatal dopa-minergic measures, and prefrontal cortex glutamatergic measures, in the same patients. Our findings, taken with those in patients at risk of psychosis,^20^ are consistent with the hypothesis that alterations in cortical glutamatergic concentrations underlie subcortical dopamine dysregulation and the development of psychotic symptoms. As above, longitudinal studies are now required to establish the direction of causality.

Previous MRS studies have either not shown a relationship between glutamate concentrations or the glutamine to glutamate ratio in the anterior cingulate and positive symptoms in early schizophrenia, or have not tested the relationship.^7^ Our finding of a direct relationship between dopamine synthesis capacity and symptoms of psychosis adds to previous findings in people with prodromal and psychotic disorders in some,^4^, ^5^ but not all studies.^30^ In studies that did not find a relationship, several factors might explain the discrepant findings. Firstly, the sample sizes in several studies were fairly modest, and they might have lacked the power to detect relationships. Second, many of them included people with chronic and stable illness, receiving antipsychotic treatment, which is likely to have reduced symptom severity.

Direct glutamatergic projections from the prefrontal cortex to the midbrain have an excitatory influence on mesostriatal dopamine neurons, and enhance dopamine release, whereas indirect pathways involving GABA-ergic interneurons have the opposite effect.^8^, ^9^ If the direct pathways play a larger role, then reduced glutamatergic activity will be accompanied by lower striatal dopaminergic signalling, whereas if indirect pathways dominate, then lower glutamatergic signalling will disinhibit mesostriatal dopamine neurons, and lead to greater dopaminergic activity. We found a negative correlation in patients, which suggests that the indirect pathway projections onto dopamine neurons pre-dominate in psychosis. However, it is important to note that it is not yet possible to distinguish the function of these projections in vivo in humans, and, thus, this is a speculative interpretation. Translational studies in animals with the same MRS and PET imaging approaches used in human studies are needed to explore the circuit level implications of our findings.

We did not find a relationship between dopamine synthesis capacity and glutamate concentrations in controls. The only other published study in healthy volunteers showed a negative relationship between striatal dopamine synthesis capacity and glutamate concentrations in the dorsolateral prefrontal cortex.^10^ This discrepancy could be due to differences in the regions of the brain investigated, as the study by Gleich and colleagues^10^ examined glutamate concentrations in the dorsolateral prefrontal cortex, whereas we investi-gated the anterior cingulate cortex. Taken together, these findings could suggest that the coupling between cortical glutamate concentration and striatal dopamine synthesis capacity is stronger in the dorsolateral prefrontal cortex than the anterior cingulate cortex.

Acute ketamine administration increases both dopamine and glutamate concentrations in animal^27^ and human studies^28^, ^29^, ^31^ although negative results have also been reported.^32^ One interpretation of these findings is that dopamine synthesis capacity and glutamate concentrations are directly related to each other, in contrast to our findings. Ketamine's acute effects on increasing dopamine concentrations are more marked in cortical regions than the striatum,^27^ whereas cortical dopamine release has been shown to be lower in patients with schizophrenia compared with controls.^33^ Thus our findings of an inverse relationship between glutamate concentration and dopamine synthesis capacity in patients with psychosis, in contrast to what is predicted from acute studies of ketamine's effects on these systems, add to the evidence that acute ketamine is not an exact model of the pathophysiology of psychosis, and highlights the need for further studies of both the dopamine and glutamate systems in people with psychosis.

In contrast to other studies,^6^, ^13^ we found no significant differences in striatal *K*_i_^cer^ or anterior cingulate glutamate concentrations between patients and controls. In addition to the possibility of a type II error, a potential reason for this difference to previous studies is that our cohort includes patients with both responsive and non-responsive forms of illness. In the responsive forms of illness, findings indicate increased dopamine concentrations and unaltered glutamate concentrations, whereas in the non-responsive forms of illness, findings indicate unaltered dopamine concentrations and increased glutamate concentrations.^15^

A strength of the current study is acquiring multimodal imaging in a comparatively large sample of people with first-episode psychosis, who were predominantly antipsychotic free or naive. Scans were taken in close temporal proximity, with a median of 6 days between scans.

A potential limitation is that abstinence from substance use before imaging was not confirmed with urine or similar drug tests for all scans, although we excluded substance dependence or abuse through clinical assessment. Thus, it is possible that recreational use of substances influenced our results; however, the effects of recreational substance use on dopamine synthesis capacity are unclear.^34^

Limitations also include the fact that three people had received a short course of treatment with antipsychotic medication. Acute treatment with a dopamine antagonist might have altered both the dopamine and glutamate measures. There is only one published longitudinal study in humans with psychosis examining the effects of antipsychotics on dopamine synthesis capacity, which suggests a decrease in dopamine synthesis capacity in the caudate and putamen with subchronic (5 weeks) haloperidol treatment.^25^ Thus, treatment could have reduced dopamine synthesis capacity in the three participants taking medication. However, cross-sectional studies in patients taking antipsychotics have shown dopamine synthesis is increased relative to controls.^3^ There is no consensus regarding the effects of dopamine blocking agents on glutamate concentrations as measured using MRS,^26^ with four of eight studies showing no significant effect of treatment on glutamate concentrations and four showing a significant decrease in Glx in left temporal lobe, frontal lobe, and thalamus, and glutamate in right striatum.^26^ Taken together, these findings suggest that if antipsychotic treatment were to influence the dopamine–glutamate relationship, it would be to reduce the inverse relationship we observe. As such, if antipsychotics are having an effect, this would not explain our findings. Moreover, the relationship between measures remained significant when medication status was accounted for in the analysis, suggesting that antipsychotic effects do not explain our findings. Nevertheless, further studies would be useful to confirm this. Although there is no in-vivo evidence of the effects of benzodiazepines and sertraline on either imaging measure, it is conceivable that these compounds had an effect, though the small number of patients taking these medictions suggests this would not markedly affect our primary findings.

It should also be recognised that the glutamate signal at 3T includes some contribution from glutamine, about 10–15%, and scanning at higher field strengths is required to more fully separate glutamate and glutamine. Further specific limitations include the relatively low spatial resolution of the imaging techniques, and the inability to differentiate intracellular and extracellular glutamate concentrations. A limitation of our study, and the field in general, is that, at the time of writing, it is not possible to delineate the precise mechanistic relationship between cortical glutamate and dopamine in vivo.

Notwithstanding the caveats discussed above, our findings suggest glutamatergic and dopaminergic systems are closely related in patients with psychosis, in line with preclinical models, and support further studies to test the effect of modulating cortical glutamate in the treatment of psychosis.
