# Corrections

**DOI:** 10.1016/S2215-0366(18)30387-0

**Published:** 2018-12

*Jauhar S, McCutcheon R, Borgan F, et al. The relationship between cortical glutamate and striatal dopamine in first-episode psychosis: a cross-sectional multimodal PET and magnetic resonance spectroscopy imaging study.* Lancet Psychiatry 2018; **5:** 816–23—In the Methods section of the Summary of this Article “…28 healthy controls…” should read “…20 healthy controls…”. This correction has been made to the online version as of Oct 1, 2018.

